# Hottest topics in hematopoietic stem cell transplantation: a summary from the 8th International Transplant and Cellular Therapy Course

**DOI:** 10.46989/001c.89034

**Published:** 2023-10-18

**Authors:** Edwin U. Suárez

**Affiliations:** 1 Hematology Department Fundación Jiménez Díaz University Hospital, Madrid, Spain

**Keywords:** Acute leukemia, Lymphoma, Multiple Myeloma, Immunoglobulin Light-chain Amyloidosis, Myeloproliferative Disorders, Myelodysplastic Syndromes, Hematopoietic Stem Cell Transplantation

The 8^th^ International Transplant and Cellular Therapy Course of the European society for Blood and Marrow Transplantation, EBMT) was held on September 8-10^th^, 2023, in Barcelona, Spain. The main topics in the field of hematopoietic progenitor transplantation and other cellular therapies were critically reviewed. Lectures on pediatric hematology were included, as well as a track focusing on treatment strategies that are of key interest for nurses. Here, we make a perspective summary of some highlights about hematopoietic stem cell transplantation (HSCT) based on the program of this meeting.

**Prophylaxis of graft-versus-host disease (GvHD).***F. Malard* (Sorbonne University, Paris, France) illustrated the prevention and treatment of acute GvHD (aGvHD). The development of the posttransplant cyclophosphamide (PTCy) strategy, thanks to the pioneers (the Baltimore group^1^), and the latest clinical trial (BMT CTN 1703^2^) has allowed the important implementation of haploidentical allogenic HSCT worldwide. Other agents are emerging, such as vedolizumab (an antibody that selectively antagonizes α4β7 gastrointestinal [GI] integrin receptors, preventing lymphocyte trafficking to the gut), which, in a phase III randomized placebo-controlled study (NCT03657160), demonstrated improved lower GI aGvHD-free survival at 180 days (presented at the 49^th^ EBMT Annual Meeting 2023). Further research is necessary to determine the optimal combinations of therapies and dosages to prevent severe aGvHD without increasing the risk of infectious complications and relapse of the underlying disease.

**Treatment of aGvHD.** First-line treatment of aGvHD remains unchanged and is based on steroids. The most effective first-line treatments for acute GvHD are still not defined, and steroid-sparing methods have become crucial because of the high failure rate and related toxicity of the former. For steroid-refractory (SR) aGvHD, ruxolitinib is the new standard (FDA and EMA approved), but around 40% of patients still fail to respond at day 28. Ruxolitinib-resistant SR aGvHD remains an unmet medical need. One of the most promising approaches is the development of gut microbiota manipulation. *F.* *Malard et al.*, recently published a comprehensive review on these subjects.^3^

**Treatment of chronic GvHD (cGvHD).** cGvHD remains the most important long-term complication of allogeneic HSCT. For the past 40 years, corticosteroids have remained the standard first-line treatment for moderate–severe cGvHD. SR cGvHD management has recently experienced modest improvement since ruxolitinib was approved for patients who failed at least one line of treatment^4^ and belumosudil (an investigational oral selective inhibitor of Rho-associated coiled-coil-containing protein kinase 2 [ROCK2], an integrator of profibrotic signals which regulates multiple profibrotic processes) for patients who failed two lines (the ROCKstar phase II clinical trial^5^). However, the ideal treatment for cGvHD remains elusive, and clear guidelines for treatment and management are lacking. Goals of therapy should focus on clinical efficacy including symptom burden reduction and prevention of progression to organ involvement by cGvHD.^4^ *O. Penack* (Charité Universitätsmedizin Berlin, Berlin, Germany) emphasized the need for better prophylactic regimens (due to the high incidence of severe cGvHD), improved drugs and/or better combinations for treatment of cGvHD (due to low complete response rates), and better prediction of treatment responses and personalized approaches (due to high non-relapse mortality, 30%–50%,^4^ due to infections and non-infectious complications).

**Cytomegalovirus (CMV).** Monitoring, cut-off points, and preventive therapy vary between different HSCT centers, as there are no studies of sufficient quality to support a particular strategy to allow for systematic conduct. *P. Lungman* (Karolinska University Hospital Huddinge, Karolinska Institutet, Stockholm, Sweden) concluded that “preemptive therapy in 2023 should still be given primarily with valganciclovir. Maribavir could be considered in 2023 in isolated patients with severe neutropenia, especially if there is also nephrotoxicity that could make treatment with foscarnet difficult. Today, we rarely need to give 2nd or 3rd line treatment for CMV; repeated valganciclovir usually works. Occasionally patients have needed foscarnet”.

**HSCT in acute myeloid leukemia (AML).** AML is the most important indication for allogeneic HSCT. *E. Brissot* (Sorbonne University, Paris, France) presented a clinical case and a practical algorithm with parameters for decision-making prior to allogeneic HSCT in patients with AML in first complete remission (CR1). This approach starts with the assessment of AML risk group classification at diagnosis (European LeukemiaNet risk stratification 2022^6^), HLA-typing, and donor availability. After induction/consolidation therapy, the quality of remission (measurable residual disease, MRD) is assessed, which is probably the second most important factor after the genetic characteristics of leukemia for defining the HSCT intervention. Subsequently, the risk of treatment-related mortality (TRM) associated with patient parameters (the hematopoietic cell transplantation - specific comorbidity index, HCT-CI or *Sorror* index^7^) and patient-donor parameters (the EBMT risk score or *Gratwohl*^8^) is determined. Considering this, allogeneic HSCT is not recommended in CR1, if patient has favorable genetics and MRD negativity after chemotherapy. Performing allogeneic HSCT in CR1 is recommended for adverse and intermediate-risk AML, but there is a grey area: MRD status after initial courses of chemotherapy dictates the transplant decision in intermediate-risk and even favorable-risk AML in CR1. Also, allogeneic HSCT should be considered in patients with relapsed or refractory AML. Targeted therapy and new agents are being incorporated as maintenance therapy for the prevention of AML relapse after allogeneic HSCT.

**HSCT in acute lymphoblastic leukemia (ALL).***S. Giebel* (Maria Sklodowska-Curie National Research Institute of Oncology, Gliwice, Poland) presented the therapeutic approach for adult patients with ALL; he showed preliminary pictures of the algorithms in ALL (with or without Philadelphia chromosome) and forthcoming is a review in the journal Clinical Hematology International, based on the most recent advances in immunotherapy (inotuzumab and blinatumomab), and new generation tyrosine kinase inhibitors (dasatinib and ponatinib) guided by the quality of response (MRD). It also discusses (without spoilers) strategies on how to optimize allogeneic HSCT for adults with ALL: donor type, conditioning, source of stem cells, and immunosuppression.

**HSCT in myelodysplastic syndromes (MDS) in the molecular era: Molecular International Prognostic Scoring System (IPSS-M).***N. Kröger* (University Hospital Eppendorf, Hamburg, Germany) discussed the steps of peri allogeneic HSCT in a clinical case of a patient with MDS. When defining the indication for transplantation, we must know the risk in terms of the disease and of the patient. For the former, we use the validated scales (revised IPSS [IPSS-R] and the current IPSS-M),^9^ thus considering the indication for HSCT at intermediate and high-risk categories. However, aspects not included in these stratifications should also be considered, such as the severity of cytopenia(s), cytogenetic features, bone marrow fibrosis, and lack of response to treatment. For the latter, we must define the patient’s functionality/fragility (with validated scales). Before the transplant, the aim of treatment will be to attenuate the disease (without this being a strict pre-transplant criterion) and improve the performance status; for this, we can reduce the tumor load (blasts) with chemotherapy, hypomethylating agents, venetoclax, which may lead to cytogenetic remission, in addition to reducing iron overload (often forgotten). The choice of conditioning will depend directly on the delicate balance between the risk of relapse (increased intensity), and the risk of MRD (reduced intensity), considering a sequential approach for upfront HSCT. Regarding donor selection, the preference, in decreasing order, should be for matched related donor (MRd) > matched unrelated donor (MUd) > haploidentical donor > mismatched unrelated donor (MMUd) / umbilical cord blood transplant. Other factors which should be considered are a young MUd instead of an older sibling; permissive mismatch; no MRd if germline mutation is present. As to GvHD prophylaxis, a calcineurin inhibitor (CNI) plus methotrexate or mycophenolate is preferred, as well as anti-thymocyte globulin (ATG) or PTCy in MUd and MRd grafts, and PTCy for haploidentical HSCT.^1,2^ Finally, one needs to prevent relapse and this must be identified early, by monitoring chimerism and/or molecular markers, lowering immunosuppression, indicating donor lymphocyte infusion (DLI), and maintenance therapy, preferably within clinical trials.

**HSCT in myeloproliferative neoplasms (MPN).***D.McLornan* (University College Hospital, London, United Kingdom) mentioned that all patients with primary myelofibrosis or post-polycythemia vera or post-essential thrombocytosis myelofibrosis should have a dynamic assessment of MPN symptomatic burden, documentation of spleen size and validated prognostic score. He also emphasized the need to discuss the cases in a multidisciplinary meeting, offer all patients a clinical trial, if available, monitor symptoms and spleen response, be vigilant for disease progression. Intermediate-2 and high/very high-risk patients, should be considered as candidates for allogeneic HSCT. If so, administering or continuing a Janus kinase inhibitor (JAKi) to maximize symptomatic response and reduce splenomegaly should be considered. In addition, he specified some details about his use of JAKi prior to allogeneic HSCT: he avoids discontinuation weeks prior to conditioning, as it may result in withdrawal symptoms. He continues low-dose ruxolitinib through conditioning until engraftment occurs. He clarified that data are scarce in this setting. In addition, his team uses letermovir as anti-CMV prophylaxis. He shared his own data showing that there appears to be no difference in survival or relapse when determining the type of conditioning intensity (myeloablative versus reduced intensity). There are several challenges in this disease, mainly in those over 65 years: the timing of allogeneic HSCT with JAKi; splenectomy prior to HSCT; choice of conditioning; integration of JAKi and others into post allogeneic HSCT conditioning/maintenance; treatment of poor graft function (role of 2nd allogeneic HSCT); relapse and role of preventive DLIs, and MRD monitoring. The discussion was enriched by a recent publication.^10^

**HCT in plasma cell neoplasms.***P. Hayden* (St. James’s Hospital, Trinity College, Dublin, Ireland) outlined the main and most current recommendations (American Society of Transplantation and Cellular Therapy, ASTCT, 2022)^11^ and studies supporting autologous-HSCT in multiple myeloma (MM) to improve mainly progression-free survival (PFS), and the controversy again raised by the results of the DETERMINATION study,^12^ which confirm no impact on overall survival (OS). This ASTCT panel^11^ continues to recommend early autologous-HSCT as consolidation therapy in eligible patients with newly diagnosed MM after 4-6 induction cycles. In addition, the panel does not recommend age as the sole selection factor when considering autologous HSCT. Nor do they recommend tandem autologous HSCT (except in the context of a clinical trial). They do recommend a second salvage autologous HSCT in patients who have been in remission for (at least) 36 months with maintenance, and 18 months in the absence of maintenance.

He also pointed out the positive impact (in terms of hematological response) of autologous HSCT in immunoglobulin-light-chain (AL) amyloidosis when patients are very carefully selected^13^ (e.g., patients with advanced cardiac amyloidosis^14^). In patients eligible for autologous HSCT, the pretransplant induction with daratumumab/cyclophosphamide/bortezomib/dexamethasone (Dara-CyBorD) or CyBorD alone is generally advised and specifically recommended for those whose bone marrow plasma cell infiltrate is >10%; if CR is reached after induction alone, HSCT can be deferred. In patients who attain a less than satisfactory response to induction and in those with concomitant MM, autologous HSCT is performed with intravenous melphalan. Patients excluded from transplantation are those with refractory orthostatic hypotension, decompensated heart failure, symptomatic and/or refractory arrhythmias, refractory and/or symptomatic pleural effusions, GI involvement with active bleeding or risk of bleeding, as well as factor X deficiency <25%.

**HSCT in autoinflammatory/autoimmune diseases (AID).** In this interesting topic, *R. Grecco* (San Raffaele Hospital, Milan, Italy) gave a comprehensive talk on the role of both autologous and allogeneic HSCT in AID,^15^ where data are scarce. With respect to autologous HSCT, it could result in clinical responses in the setting of patients with inflammatory activity and refractory to other therapies, but further studies are undoubtedly required to establish the best autologous HSCT regimen and the relative benefit over current or future disease-modifying therapies. On the other hand, allogeneic HSCT may be potentially curative, but the role in AID needs to be defined, as well as the best conditioning and prophylaxis against GvHD.

**HSCT in sickle cell disease (SCD) and transfusion dependent thalassemia (TDT).** Dr. *S. Corbacioglu’s* (University of Regensburg, Regensburg, Germany) keynote lecture discussed the transition from allogeneic HSCT to gene therapy for the most common hemoglobinopathies. Among the absolute indications for HSCT, he mentioned disease-related complications that cannot be avoided with conventional treatment (essentially the case for most patients). Moreover, the results of haploidentical HSCT (PTCy) are increasingly comparable to those with a matched sibling donor / MUd HSCT, with excellent OS and quality of life. Late HSCT (in adults) is also feasible but must be well prepared, with aggressive reduction of iron overload (mainly in TDT). Regarding risks, he mentioned mainly transplant-associated vasculopathy (veno-occlusive disease/sinusoidal obstruction syndrome) and reversible posterior encephalopathy syndrome, stroke, and macrophage activation syndrome in haploidentical HSCT. Early rejection due to hyper-transfusion, donor-specific antibodies, and AB0 mismatch, are among the main ones.

**HSCT in lymphoproliferative diseases.***Anna Sureda* (Institut Català d’Oncologia-L’Hospitalet, Instituto de Investigación Biomédica de Bellvitge, Universitat de Barcelona, Barcelona, Spain) discussed the current role of transplantation in aggressive B-cell lymphomas (mainly diffuse large B-cell lymphoma, DLBCL), after the advent of autologous CAR-T cell therapy. She mentioned that, currently (its most recent publication in 2022^15^), the EBMT recommends prioritizing autologous CAR-T cell therapy as the standard of care (SOC) in patients who have failed 2 or more lines of treatment, which will continue the trend in the decreasing number of allogeneic HSCTs. However, autologous HSCT remains the SOC for late chemosensitivity relapses in DLBCL and primary central nervous system lymphoma. Other scenarios worth mentioning are chemo-sensitive relapsed Hodgkin disease and CR1 mantle cell lymphoma as SOC indications for autologous HSCT.

## Conflicts of interest

The author declares he has no conflicts of interest.

## Data availability

Not applicable.
